# Pro-inflammatory cytokines in cryptoglandular anal fistulas

**DOI:** 10.1007/s10151-016-1494-7

**Published:** 2016-07-11

**Authors:** R. S. van Onkelen, M. P. Gosselink, M. van Meurs, M. J. Melief, W. R. Schouten, J. D. Laman

**Affiliations:** 1 Department of Surgery, Erasmus Medical Center Rotterdam, University Medical Center, Room H 181’s Gravendijkwal 230, 3015 CE Rotterdam, The Netherlands; 2 Department of Immunology, Erasmus MC, University Medical Center, MS Center ErasMS, Rotterdam, The Netherlands; 3 Department of Surgery, Maasstad Hospital, Rotterdam, The Netherlands; 4 Department of Neuroscience, University Medical Center, University of Groningen, Groningen, The Netherlands

**Keywords:** Perianal fistulas, Platelet-rich plasma, Auto-inflammatory, Immunology, Bacteria, Microbiota

## Abstract

**Background:**

Sphincter-preserving procedures for the treatment of transsphincteric fistulas fail in at least one out of every three patients. It has been suggested that failure is due to ongoing disease in the remaining fistula tract. Cytokines play an important role in inflammation. At present, biologicals targeting cytokines are available. Therefore, detection and identification of cytokines in anal fistulas might have implications for future treatment modalities. The objective of the present study was to assess local production of a selected panel of cytokines in anal fistulas, including pro-inflammatory interleukin (IL)-1β and tumor necrosis factor α (TNF-α).

**Methods:**

Fistula tract tissue was obtained from 27 patients with a transsphincteric fistula of cryptoglandular origin who underwent flap repair, ligation of the intersphincteric fistula tract or a combination of both procedures. Patients with a rectovaginal fistula or a fistula due to Crohn’s disease were excluded. Frozen tissue samples were sectioned and stained using advanced immuno-enzyme staining methods for detection of selected cytokines, IL-1β, IL-8, IL-10, IL-12p40, IL-17A, IL-18, IL-36 and TNF-α. The presence and frequencies of cytokine-producing cells in samples were quantitated.

**Results:**

The key finding was abundant expression of IL-1β in 93 % of the anal fistulas. Frequencies of IL-1β-producing cells were highest (>50 positive stained cells) in 7 % of the anal fistulas. Also, cytokines IL-8, IL-12p40 and TNF-α were present in respectively 70, 33 and 30 % of the anal fistulas.

**Conclusions:**

IL-1β is expressed in the large majority of cryptoglandular anal fistulas, as well as several other pro-inflammatory cytokines.

## Introduction

In a previous study, conducted in patients with a persistent anal fistula after flap repair, we observed complete healing of the flap, except at the site of the original internal fistula opening [1]. This observation and the fact that there are, most likely, no factors predisposing to failure suggest that persistence of anal fistulas is caused by ongoing inflammation in the remaining fistula tract. A recent study, conducted at our institution, revealed that the majority of anal fistulas are lined with granulation tissue (75 %), which is suggestive of an inflammatory response [2].

Since local bacterial replication might be an inflammatory factor, we performed another study [3]. A paucity of bacteria was observed in the fistula tracts. Bacterial species were bowel derived, skin derived or a combination of both. No mycobacterium species were identified. Therefore, it seems unlikely that bacterial infection plays a major role in persistence of fistula after surgery. These results were recently confirmed by Tozer et al. [4].

In the same study, we detected pro-inflammatory peptidoglycan in the majority of anal fistulas [3]. Peptidoglycan is a major component of the bacterial cell wall of both Gram-positive and Gram-negative species, providing structural strength and allowing bacteria to resist osmotic pressure. Peptidoglycan has potent pro-inflammatory properties and stimulates processing and secretion of the cytokine interleukin (IL)-1β [5, 6]. This suggests that even in the absence of local bacterial replication, bacterial components promote chronic inflammation. Studies on the pathogenesis of different chronic inflammatory diseases, such as Crohn’s disease, show that peptidoglycan is a powerful effector-stimulating inflammation [7, 8].

Cytokines play an important role in inflammation. Tumor necrosis factor (TNF)-α, IL-1β and other cytokines promote inflammatory responses that cause many of the clinical problems associated with immune-mediated diseases, such as rheumatoid arthritis, hidradenitis suppurativa, ulcerative colitis and Crohn’s disease [9–12]. Biologicals targeting cytokines, such as anti-TNF-α and IL-1 receptor antagonist, are used in various diseases and clinical trials [13]. An overview of these agents is presented in Table 1, focusing on cytokines addressed in the current study. The question is whether one of these agents might play a role as adjunct in the treatment of anal fistulas.

**Table 1:** Summary of cytokines assessed and therapeutic options

Cytokine	Main functions	Therapeutic molecules (generic name of biological^a^)	Main (potential) clinical applications
IL-1β	Inflammatory response to infection. Mediates fever and promotes formation of acute phase proteins by the liver. Activation of T and B lymphocytes. Elevates adhesion molecules on endothelium. Induces other cytokines such as IL-6. Resembles TNF-α in its inflammatory properties	Anakinra (IL-1 receptor antagonist); rilonacept (IL-1 receptor fusion protein); canakinumab (anti-IL-β mab)	RA, CAPS such as Muckle–Wells syndrome (MWS) and familial cold auto-inflammatory syndrome (FCAIS)
IL-8 alias CXCL8	Mediator of innate immune responses. Induces chemotaxis of T cells and neutrophilic granulocytes. Promotes phagocytosis and angiogenesis	Limited	Spectrum of (auto)inflammatory diseases
IL-10	Anti-inflammatory. Inhibits production of cytokines by many cell types. Inhibits activation and effector functions of T cells, monocytes and macrophages	Limited, e.g., Tenovil (recombinant IL-10)	Spectrum of (auto)inflammatory diseases
IL-12p40	Differentiation of naive T cells into T helper 1 cells. Induction of IFN-γ in NK cells and T cells. Growth factor for activated CD4 + and CD8 + T lymphocytes and NK cells, and enhancement of their function	Ustekinumab (mab against shared p40 subunit of IL-12 and IL-23); briakinumab (mab against shared p40 subunit of IL-12 and IL-23)	Psoriasis, CD
IL-17A	Central to induction and maintenance of pro-inflammatory responses. Induces other inflammatory cytokines and mediators, notably chemoattractants including those for neutrophilic granulocytes. IL-17A is critical to function of the Th17 subset of CD4 + lymphocytes	Secukinumab (mab against IL-17A); ixekizumab (mab against IL-17A); brodalumab (mab against IL-17 receptor)	Psoriasis, RA, AS, RRMS
IL-18 (IL-1 family)	Induces cell-mediated immunity to intracellular pathogens. Induces IFN-γ, and other cytokines and chemokines	Limited	Spectrum of (auto)inflammatory diseases
IL-36 (IL-1 family)	Acts directly on naive T cells, enhancing proliferation and IL-2 production. Stimulates Th1 responses. Acts on dendritic cells. Appears to be especially expressed and functional in the skin	Limited	Spectrum of (auto)inflammatory diseases
TNF-α	Participates in inflammation, wound healing and remodeling of tissue. Induces apoptosis, other cytokines and inflammation. Facilitates leukocyte recruitment, induces angiogenesis and promotes fibroblast proliferation. Induces expression of adhesion molecules on vascular endothelium	Infliximab (anti-TNF mab); adalimumab (anti-TNF mab); golimumab (anti-TNF mab); etanercept (soluble receptor for TNF); certolizumab (anti-TNF mab)	RA, CD, UC, AS, PsA, JIA, Psoriasis, HS

*AS* ankylosing spondylitis, *CAPS* cryopyrin-associated periodic syndromes, *CD* Crohn’s disease, *HS* hidradenitis suppurativa, *IL* interleukin, *JIA* juvenile idiopathic arthritis, *mab* monoclonal antibody, *NK* natural killer, *PsA* psoriatic arthritis, *RA* rheumatoid arthritis, *RRMS* relapsing remitting multiple sclerosis, *TNF* tumor necrosis factor, *UC* ulcerative colitis [14–17]

^a^Nomenclature of biologicals includes: -cept for receptor, -ki(n) for interleukin, -mab for monoclonal antibody, -ra for receptor antagonist, -u for human, -zu for humanized

The objective of the present observational study was to detect and to identify cytokines in anal fistulas of cryptoglandular origin.

## Materials and methods

### Study design

Anal fistula tissue was obtained from 27 patients with a transsphincteric fistula of cryptoglandular origin that underwent transanal advancement flap repair (TAFR), ligation of the intersphincteric fistula tract (LIFT) or a combination of both procedures at the Division of Colon and Rectal Surgery, Erasmus MC, University Medical Center. Prior to the procedure, patients underwent endoanal magnetic resonance imaging to visualize the course of the fistula tract and to determine the presence and location of associated abscesses. Patients with a rectovaginal fistula and/or a fistula due to Crohn’s disease were excluded from this study. None of the patients had hepatitis B and/or human immunodeficiency virus (HIV) infection at the time of surgery. None of the patients used antibiotics and/or immunomodulatory drugs prior to surgery. Baseline patient and fistula characteristics are presented in Table 2. All patients provided informed consent meeting the standards set by the hospital’s institutional review board. All operations were performed in a time period of 3 years by one surgeon and several colorectal surgery fellows. Patients were treated in a day-care setting.

**Table 2:** Baseline patient and fistula characteristics (*N* = 27)

Patient and/or fistula characteristic	*N*
Age at surgery (years)	46.2 ± 10.5
Sex (male)	15 (55.6)
Fistula type	
HTS	22 (81.5)
LTS	5 (18.5)
Previous attempts at repair	
0	8 (29.6)
≥1	18 (66.7)
Location internal fistula opening^a^	
Anterior	10 (37.0)
Posterior	15 (55.6)
Lateral	1 (3.7)
Location external fistula opening	
Anterior	0 (0)
Posterior	4 (14.8)
Lateral	23 (85.2)
Type of operative technique	
TAFR	13 (48.2)
LIFT	6 (22.2)
TAFR and LIFT	8 (29.6)

Categorical variables are presented as numbers (%). Continuous values are expressed as mean ± SD

*HTS* high transsphincteric fistula, *LTS* low transsphincteric fistula, *TAFR* transanal advancement flap repair, *LIFT* ligation of intersphincteric fistula tract

^a^The internal fistula opening was not identified in one patient

### Operative techniques

We have described our techniques of TAFR and LIFT earlier in detail [18].

### Sample collection

The external fistula opening was enlarged, and the fistula tract was excised as far as possible until the outer border of the external anal sphincter. Immediately after excision, the fistula tract was frozen using dry ice and transported to the laboratory for cryopreservation. Excised tissue was cryopreserved in liquid nitrogen and subsequently stored at −80 °C for processing. The value of samples from healthy controls or patients with other diseases as reference groups has been considered. However, the only possibility to obtain healthy anal tissue (including anal glands) from patients is during major surgery of this specific part of the body. Indications for these types of surgery are Crohn’s disease or colorectal cancer. These diseases would certainly bias any findings as they are associated with increased expression of cytokines [11, 19]. Also, the potential value of samples from deceased patients without colorectal diseases as reference groups has been considered. However, we reasoned that any cause of death and death itself might probably bias any findings. Therefore, we have decided to use no reference group.

### In situ analysis of cytokine-producing cells

Frozen tissue samples were sectioned and stained using advanced immuno-enzyme staining methods for detection of selected cytokines IL-1β, IL-8, IL-10, IL-12p40, IL-17A, IL-18, IL-36 and TNF-α. This selection of cytokines was designed based on current concepts of tissue inflammation versus inflammation control (e.g., IL-10) and the availability of clinically approved biologicals (see Table 1). In short, frozen sections of 6 μm in thickness were dried overnight in a humidified box before getting fixed with freshly prepared acetone containing 0.02 % of hydrogen peroxide to inhibit endogenous peroxidase activity by cells in the tissue (e.g., granulocytes) for 10 min. Histochemical revelation of endogenous peroxidase with 4-chloro-1-naphthol was performed, resulting in a dense blue-black precipitate. After this, the sections were incubated overnight with primary antibodies at previously determined optimal dilutions at 4 °C. The following commercially available antibodies were used: antihuman IL-1β (clone 8516.31, R&D Systems, Minneapolis, MN, USA), antihuman IL-8 (clone G265-8, BD Biosciences Pharmingen, San Jose, CA, USA), antihuman IL-10 (clone JES3-12G8, BD Biosciences Pharmingen, San Jose, CA, USA), antihuman IL-12p40 (clone C8.6, BD Biosciences Pharmingen, San Jose, CA, USA), antihuman IL-17A (clone e B 1064 CAP, eBioscience Inc., San Diego, CA, USA), antihuman IL-18 (rabbit IgG, Thermo Scientific, Rockford, IL, USA), antihuman IL-36 (clone 3A12, Abcam, Cambridge, MA, USA) and antihuman TNF-α (clone 61E7, U-CyTech Biosciences, Utrecht, The Netherlands). Secondary enzyme-labeled antibodies were applied and incubated at room temperature. Between incubations the slides were rinsed twice in phosphate-buffered saline/0.05 % Tween 20. Histochemical revelation of horseradish peroxidase activity was performed using aminoethyl carbazole (AEC, Sigma-Aldrich, St. Louis, MO, USA) for a bright red translucent staining. Finally, sections were counterstained with hematoxylin and mounted in Kaiser’s glycerin gelatin with a cover slip. Sections of reactive human tonsils were used as internal positive control tissue since they contain numerous leukocytes producing a variety of cytokines. Staining controls were done by omission of the primary antibody and by isotype- and subclass-matched negative control antibodies.

### Quantitation of cytokine-producing cells

In order to interpret the presence and frequencies of cytokine-producing cells, the number of positive stained cells in the fistula samples was scored from zero to four: zero for no stained cells, one for 1–5 stained cells, two for 6–20 stained cells, three for 21–50 stained cells and four for >50 stained cells. All sections were evaluated by two independent observers.

### Statistical analysis

Continuous data are presented as mean values with standard deviation. Categorical data are presented as frequencies or percentages. Data were analyzed by use of SPSS-IBM^®^ software version 20.0 for Windows^®^ (SPSS, Chicago, IL, USA).

## Results

**Fig. 1:**
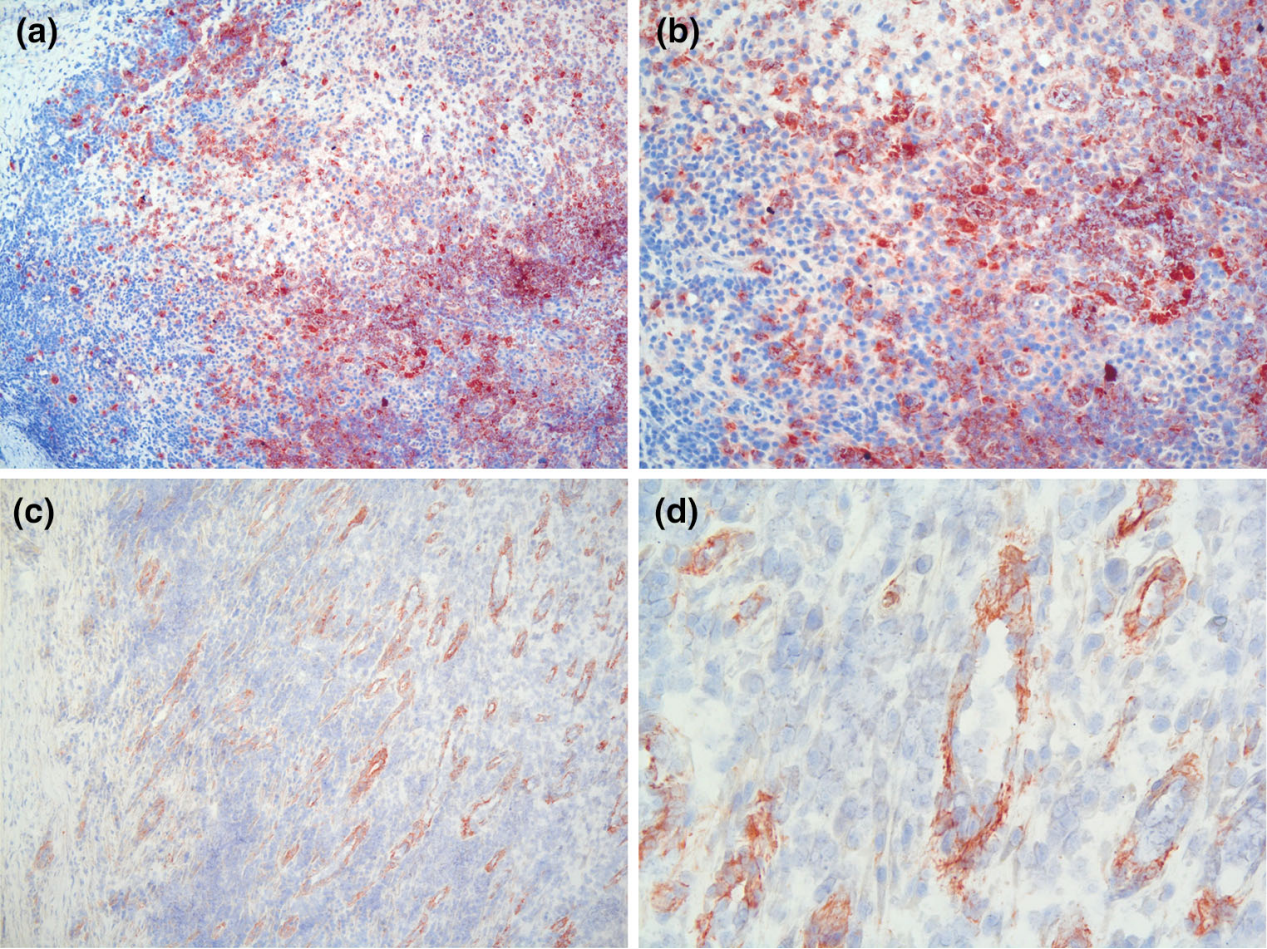
IL-1β and IL-8 in anal fistula samples. IL-1β and IL-8 are expressed by cells within infiltrates of the anal fistula tissue. The presence of IL-1β (*red*) and IL-8 (*red*) was detected by specific antibodies. IL-1β is presented in the **a** (magnification 100*×*) and **b** (magnification 200*×*). IL-8 is presented in the **c** (magnification 100*×*) and **d** (magnification 400*×*)

Anal fistula samples were obtained from 27 patients with a transsphincteric fistula of cryptoglandular origin during surgery. Of these, 13 patients (48 %) underwent flap repair, 6 patients (22 %) underwent ligation of the intersphincteric fistula tract (LIFT) and 8 patients (30 %) underwent a combination of both procedures. The majority of the patients had undergone a previous attempt at repair (67 %). Based on available information, half of the patients consumed alcohol and one-third of the patients smoked. Baseline patient and fistula characteristics are presented in Table 2. The presence and frequencies of cytokine-producing cells in anal fistula samples were evaluated using a pathology scoring system. These findings are presented in Table 3. In the positive control tissue, reactive human tonsils from children, cells producing all cytokines were detected in varying numbers, as predicted. This confirms the anticipated reactivity of the antibodies used and their suitability for detection of cytokines in frozen tissue. In 93 % of the fistula samples, the prominent pro-inflammatory cytokine IL-1β was present. Frequencies of IL-1β-producing cells were highest (>50 positive stained cells) in 7 % of the fistula samples (Fig. 1). The cytokines IL-8, IL-12p40 and TNF-α were present in 70, 33 and 30 %, respectively, of the fistula samples (Fig. 1). IL-10, IL-17A, IL-18 and IL-36 could not be detected. No significance difference in cytokine expression was detected between patients with a persistent fistula after previous attempts at repair and those presenting with a new fistula. However, most likely the sample sizes are too small to detect any differences between the groups.

**Table 3:** Presence and frequencies of cytokine-producing cells in anal fistulas (*N* = 27)

Cytokine	*N* (%)	No stained cells	1–5 Stained cells	6–20 Stained cells	21–50 Stained cells	>50 Stained cells
IL-1β	25 (92.6)	2 (7.4)	4 (14.8)	12 (44.4)	7 (26.0)	2 (7.4)
IL-8	19 (70.4)	8 (29.7)	4 (14.8)	6 (22.2)	5 (18.5)	4 (14.8)
IL-10	0 (0)	27 (100)	0 (0)	0 (0)	0 (0)	0 (0)
IL-12p40	9 (33.3)	18 (66.7)	1 (3.7)	7 (25.9)	1 (3.7)	0 (0)
IL-17A	0 (0)	27 (100)	0 (0)	0 (0)	0 (0)	0 (0)
IL-18	0 (0)	27 (100)	0 (0)	0 (0)	0 (0)	0 (0)
IL-36	0 (0)	27 (100)	0 (0)	0 (0)	0 (0)	0 (0)
TNF-α	8 (29.6)	19 (70.4)	3 (11.1)	4 (14.8)	1 (3.7)	0 (0)

Variables are presented as numbers (%)

*IL* interleukin, *TNF* tumor necrosis factor

## Discussion

The key finding of the present study was the abundant expression of pro-inflammatory cytokine IL-1β in 93 % of the anal fistulas. Also, the cytokines IL-8, IL-12p40 and TNF-α were expressed in a high number of anal fistulas (Table 3).

Limitations of this study are the lack of a control or reference group, the small number of patients, lack of additional staining for identification of cell types that produce cytokines and a potential selection bias caused by the role of our institution as tertiary referral center. Strengths of the study are the comprehensive panel of cytokines studied and the homogeneity of the patient group.

IL-1 is a central mediator of innate immunity, inflammation and fever. As a highly active pro-inflammatory cytokine, it contributes to lowering pain thresholds and to tissue damage. IL-1β can be produced by many different cell types, with a prominent role of monocytes and macrophages [20]. The stimulus can be microbial products, cytokines and even IL-1β itself [21]. Self-induction by IL-1 is part of the mechanism of auto-inflammation [12]. The IL-1β precursor is inactive within the cell, and biologically active IL-1β is produced by inflammasomes that cleave pro-IL-1β using caspase-1.

Based on our previous study, it seems likely that peptidoglycan is a one of the potential stimuli for the expression of IL-1β in anal fistulas. Peptidoglycan induces inflammasome NLRP3-mediated caspase-1 activation and thereby processing and secretion of IL-1β. This concept is supported by the presence of peptidoglycan in 90 % of cryptoglandular anal fistulas [3]. Self-induction by IL-1 may also be involved.

Overall, our findings suggest that cytokines contribute to the inflammatory process in cryptoglandular anal fistulas.

Flap repair has been advocated as the treatment of choice for high transsphincteric anal fistulas and enables healing in two of every three patients [22, 23]. A pilot study by Verhagen et al. showed that flap repair with additional injection of platelet-rich plasma (PRP) in the anal fistula tract increases the healing rate to 90 % [24]. A second study with a longer duration of follow-up confirmed these promising results [25]. According to the authors, PRP improves wound healing and may therefore improve the closure rate of anal fistulas. PRP has also anti-inflammatory potentials. Kim et al. [26] showed that PRP is able to suppress expression of degrading enzymes and mediators induced by TNF-α and IL-1β. It seems likely that this mode of action also contributes to the higher healing rate after flap repair. Currently, three IL-1 antagonists (anakinra, rilonacept and canakinumab) targeting IL-1β are approved for treatment of various diseases (Table 1). Studies on IL-1 antagonists targeting a broad spectrum of new indications, such as hidradenitis suppurativa, show promising results [27].

## Conclusions

Pro-inflammatory IL-1β is expressed in the large majority of cryptoglandular anal fistulas. The clinical value of these findings must be further studied before we can proceed to apply adjunct treatment of anal fistulas by targeting cytokines.
